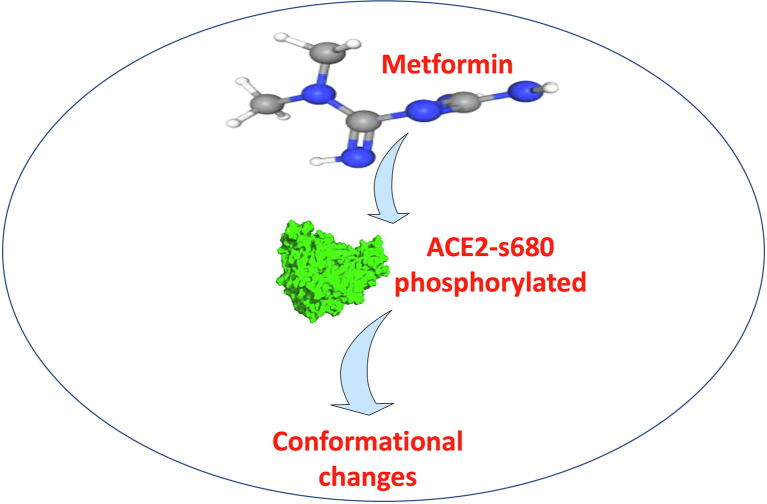# Molecular dynamic and bioinformatic studies of metformin-induced ACE2 phosphorylation in the presence of different SARS-CoV-2 S protein mutations

**DOI:** 10.1016/j.sjbs.2023.103699

**Published:** 2023-06-03

**Authors:** Amr Ahmed El-Arabey, Mohnad Abdalla, Adel Rashad Abd-Allah, Hanin.S. Marenga, Yosra Modafer, Abeer S. Aloufi

**Affiliations:** a Department of Pharmacology and Toxicology, Faculty of Pharmacy, Al-Azhar University, Cairo 11751, Egypt; b Pediatric Research Institute, Children’s Hospital Affiliated to Shandong University, Jinan 250022, China; c Department of Pharmaceutical chemistry, College of pharmacy, King Saud University, Riyadh 11451, Saudi Arabia; d Department of Biology, Faculty of Science, Jazan university, Jazan 45142, Saudi Arabia; e Department of Biology, College of Science, Princess Nourah bint Abdulrahman University, P.O. Box 84428, Riyadh 11671, Saudi Arabia

**Keywords:** SARS-CoV-2, COVID-19, ACE2, B^0^AT1, Metformin, ACE2- phosphorylation at s680

## Abstract

The SARS-CoV-2 infection activates host kinases and causes high phosphorylation in both the host and the virus. There were around 70 phosphorylation sites found in SARS-CoV-2 viral proteins. Besides, almost 15,000 host phosphorylation sites were found in SARS-CoV-2-infected cells. COVID-19 is thought to enter cells via the well-known receptor Angiotensin-Converting Enzyme 2 (ACE2) and the serine protease TMPRSS2. Substantially, the COVID-19 infection doesn’t induce phosphorylation of the ACE2 receptor at Serin-680(s680). Metformin's numerous pleiotropic properties and extensive use in medicine including COVID-19, have inspired experts to call it the “aspirin of the twenty-first century”. Metformin's impact on COVID-19 has been verified in clinical investigations via ACE2 receptor phosphorylation at s680. In the infection of COVID-19, sodium-dependent transporters including the major neutral amino acid (B^0^AT1) is regulated by ACE2. The structure of B^0^AT1 complexing with the COVID-19 receptor ACE2 enabled significant progress in the creation of mRNA vaccines. We aimed to study the impact of the interaction of the phosphorylation form of ACE2-s680 with wild-type (WT) and different mutations of SARS-CoV-2 infection such as delta, omicron, and gamma (γ) on their entrance of host cells as well as the regulation of B^0^AT1by the SARS-CoV-2 receptor ACE2. Interestingly, compared to WT SARS-CoV-2, ACE2 receptor phosphorylation at s680 produces conformational alterations in all types of SARS-CoV-2. Furthermore, our results showed for the first time that this phosphorylation significantly influences ACE2 sites K625, K676, and R678, which are key mediators for ACE2-B^0^AT1 complex.

## 1 Introduction

In China, a severe acute respiratory illness caused by coronavirus emerged in 2002. Ten years later, in 2012, a new strain of the Middle East Respiratory Syndrome Coronavirus virus emerged in the Middle East. The Chinese Center for Disease Control and Prevention (China CDC) reported pneumonia of unknown origin at the end of 2019. The pneumonia was connected to a wet animal and seafood wholesale market in Wuhan, Hubei Province, China, and was eventually identified as COVID-19. The current worldwide coronavirus disease 2019 (COVID-19) epidemic is caused by severe acute respiratory syndrome coronavirus 2. In this way, COVID-19 has presented enormous hurdles to healthcare systems all around the world since its discovery (El-Arabey and Abdalla 2020). COVID-19 has affected more than 676 million people worldwide and caused 6,881,955 deaths in 200 countries as of March 10, 2023 https://coronavirus.jhu.edu/map. Several investigations have concluded that COVID-19 enters cells via the well-known receptor Angiotensin-Converting Enzyme 2 (ACE2) and the serine protease TMPRSS2. The interaction of ACE2 with the SARS-CoV-2 spike protein is essential for viral entry and subsequent infection (Pique-Regi et al., 2020, El-Arabey and Abdalla, 2021). ACE2 is largely expressed by epithelial cells of the lung, stomach, kidney, and blood vessels. This might explain why persons with severe COVID-19 infections are more likely to develop pneumonia and bronchitis. According to a recent study, ACE2 is also highly expressed in the mucosa of the oral cavity, allowing the virus to infect a new susceptible host (Ciaglia et al., 2020). As a result, blocking this binding event or reducing the virus's accessibility to the ACE2 receptor is an alternative technique for avoiding COVID-19. Furthermore, ACE2′s biological significance in regulating the innate immune system and tissue repair pathways underlines its therapeutic potential in treating infected persons(Jia et al., 2021). Preclinical data shows that after SARS-CoV-2 binding, ACE2 may be downregulated, and treatments that increase ACE2 may protect against cardiopulmonary injury(Malhotra et al., 2020). According to recent research, monitoring plasma ACE2 may be effective in predicting COVID-19 outcomes. Furthermore, ACE2 is linked to the severity of COVID-19 illness and known risk factors such as hypertension, pre-existing heart disease, and pre-existing renal disease(Kragstrup et al., 2021, Abdalla and Rabie, 2023). It suggests that ACE2 is involved in the link between diabetes and COVID-19. In people with diabetes, ACE2 expression is reduced, most likely due to glycosylation; this might explain the increased risk of severe lung injury and respiratory distress syndrome with COVID-19(Pal and Bhansali 2020).

Metformin was found in the 1940 s while looking for antimalarial drugs and proved beneficial in treating influenza during a clinical trial by reducing blood glucose. Metformin was licensed by the Food and Drug Administration (FDA) in 1995 as an oral hypoglycemic agent, and it has since become one of the most commonly prescribed diabetes medications in the world, with the potential for further therapeutic applications(El-Arabey and Abdalla 2020). Several studies in recent years have highlighted metformin's potential usefulness as a promising medicine for treating polycystic ovarian syndrome, cancer, aging, cardiovascular disease, metabolic syndrome, and neurological illnesses. Furthermore, it is used off-label for weight loss in the United States(Wang et al., 2016, El-Arabey, 2018). Metformin appears to have unique activities in treating autoimmune illness, including decreased macrophage cytokine production. Furthermore, it has been proposed that metformin may suppress the virus by improving insulin sensitivity(El-Arabey and Abdalla 2020). According to many observational retrospective investigations, metformin users had a lower mortality rate than non-users. Metformin's impact may be attributed to numerous mechanisms, including improved glucose regulation, increased cellular pH, a decrease of (body weight, insulin resistance, neutrophils, mitochondrial reactive oxygen species, inflammatory mediators), and blockage of the mTOR pathway(Scheen 2020). Furthermore, metformin acts as an AMPK activator, causing ACE2 phosphorylation at s680(Varghese et al., 2021, Eltayb et al., 2023). Besides, it was proposed theoretically that metformin may decrease the penetration of SARS-CoV-2 to the host cells(Scheen 2020).

The ACE2 enhances sodium-dependent transporters, including the major neutral amino acid (B^0^AT1) trafficking to the plasma membrane and modulates transporter activity in the oocyte expression system(Loo and Wright 2021). Aside from ACE2′s role as the SARS-CoV-2 receptor and the necessity of co-expressing B^0^AT1 with ACE2 in creating a COVID-19 mRNA vaccine, the 3D atomic coordinates prompted intriguing issues concerning ACE2 and B^0^AT1′s functional connection. Such inquiries include how ACE2 regulates B^0^AT1 brush border expression and activity in the intestine(Loo and Wright 2021). Based on the prior findings, there is reason to believe that metformin is a viable adjuvant therapy with routine hospital care in hospitalized patients with Severe Acute Respiratory Syndrome related to SARS-CoV2 with or without diabetes. Hence, the purpose of this study is to assess the molecular dynamic effect of ACE2-s680 phosphorylation by metformin on the interaction with wild-type (WT) and different forms of COVID-19 mutations such as Delta (δ), omicron and gamma (γ) as well as the SARS-CoV-2 receptor ACE2′s control of B^0^AT1.

## 2 Methodology

### 2.1 MD simulation

The PDB file 7DF4(Wang et al., 2021) was used as WT SARS-CoV-2 S protein. The MD simulation was run on Desmond with default protocols(Bowers et al., 2006). The TIP3P model in Desmond System Builder tool was used to solvate the protein. Periodic boundary conditions with 10 Å orthorhombic box were used on the outer protein surface, and 0.15 M NaCl was used to neutralize the simulation system. The simulation was run at temperature of 310 K and pressure of 1.013 bar for 100 ns. The trajectory was analyzed by Desmond, VMD(Humphrey et al., 1996), and PyMOL.

### 2.2 Bioinformatics

Using cutting-edge high-throughput omics methods, we investigated COVID-19 infection from a variety of viewpoints linked to phosphorylation and ACE2. The COVINET database includes basic knowledge of SARS-CoV2, as well as aiding in the development of antiviral drugs(Stukalov et al., 2021).

## 3 Results

### 3.1 SARS-CoV2 induces phosphorylation at numerous genes including ACE2, which varies over time

**Fig. 1:**
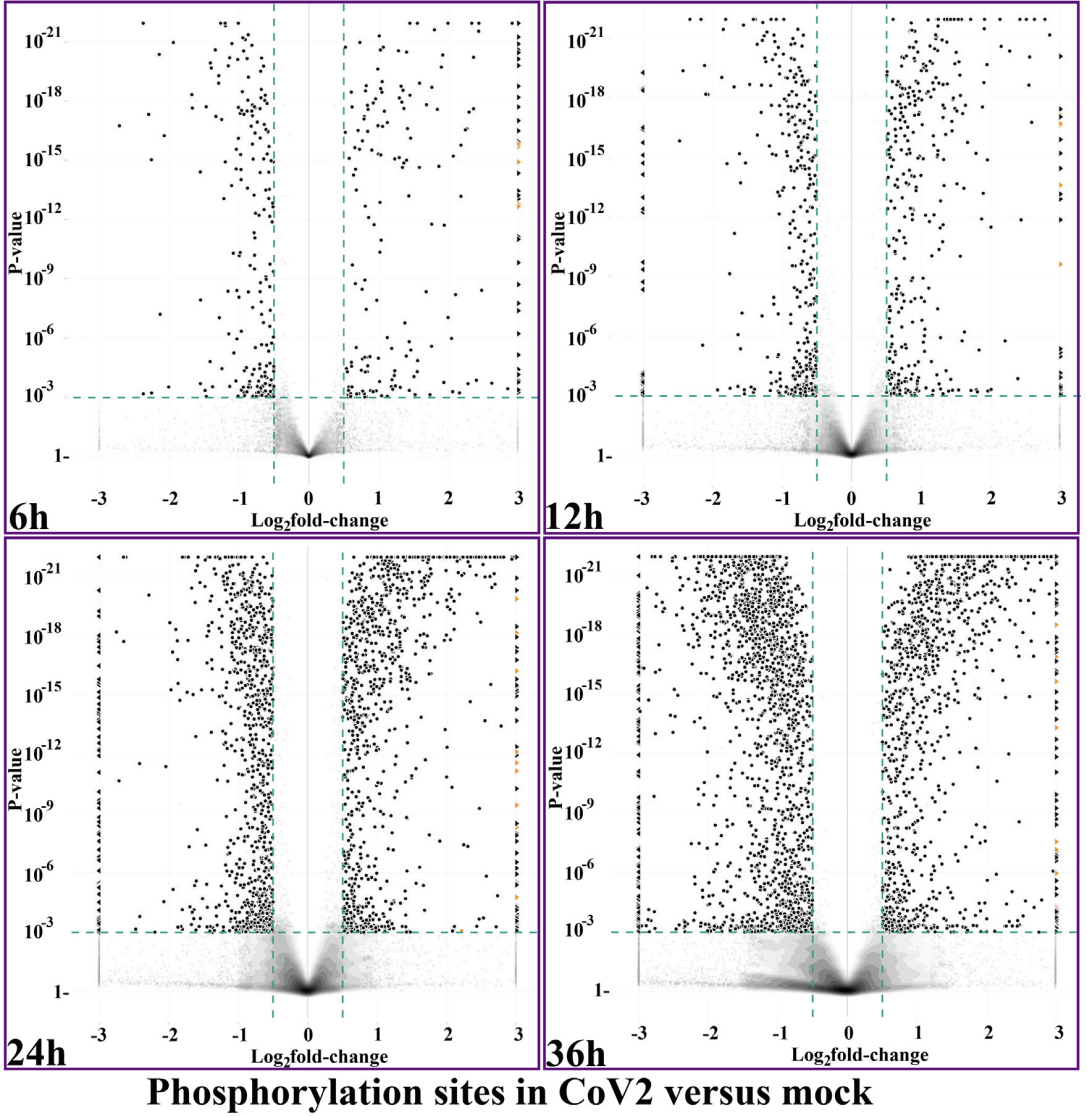
Phosphorylation sites in SATS-CoV2 versus mock after 6,12,24, and 36 h.

**Fig. 2:**
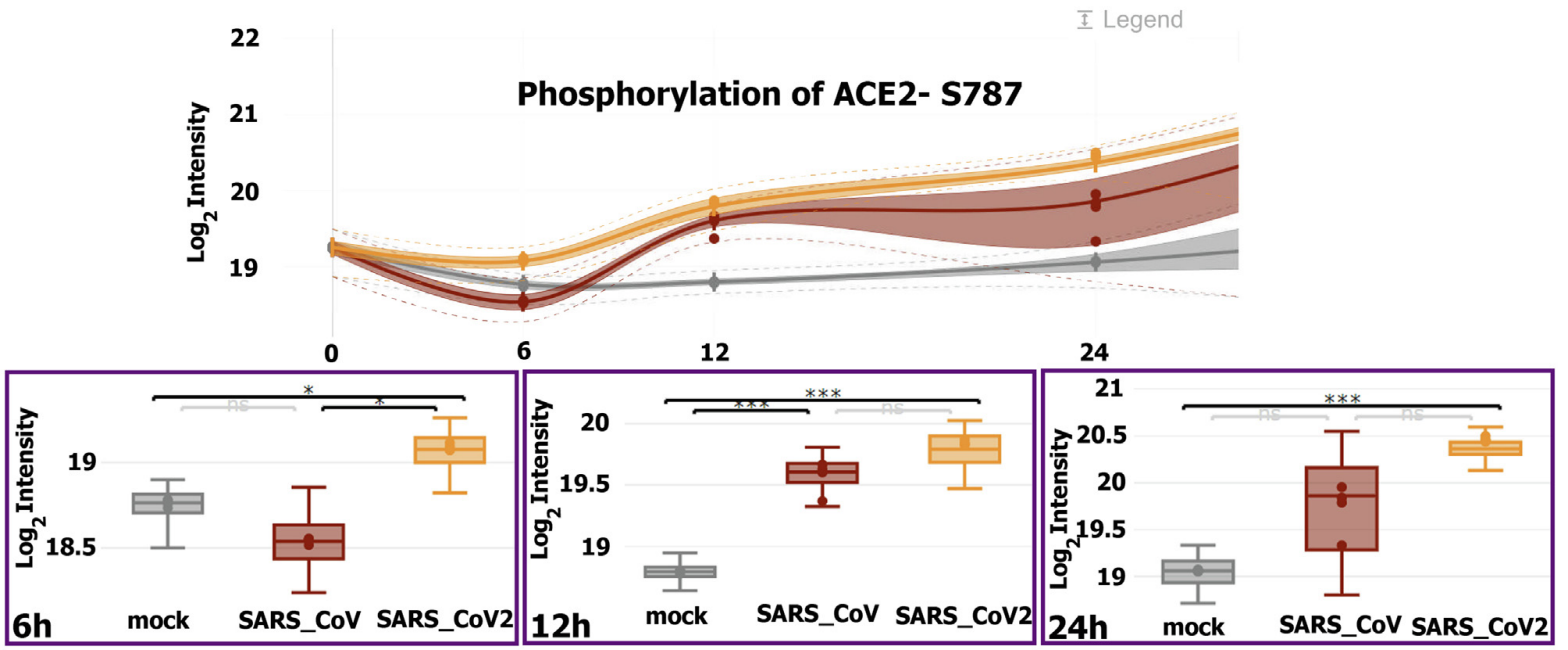
Intensity of phosphorylation of ACE2-S787 in mock, SARS-COV, and SARS-CoV2 after 6, 12, and 24 h.

**Fig. 3:**
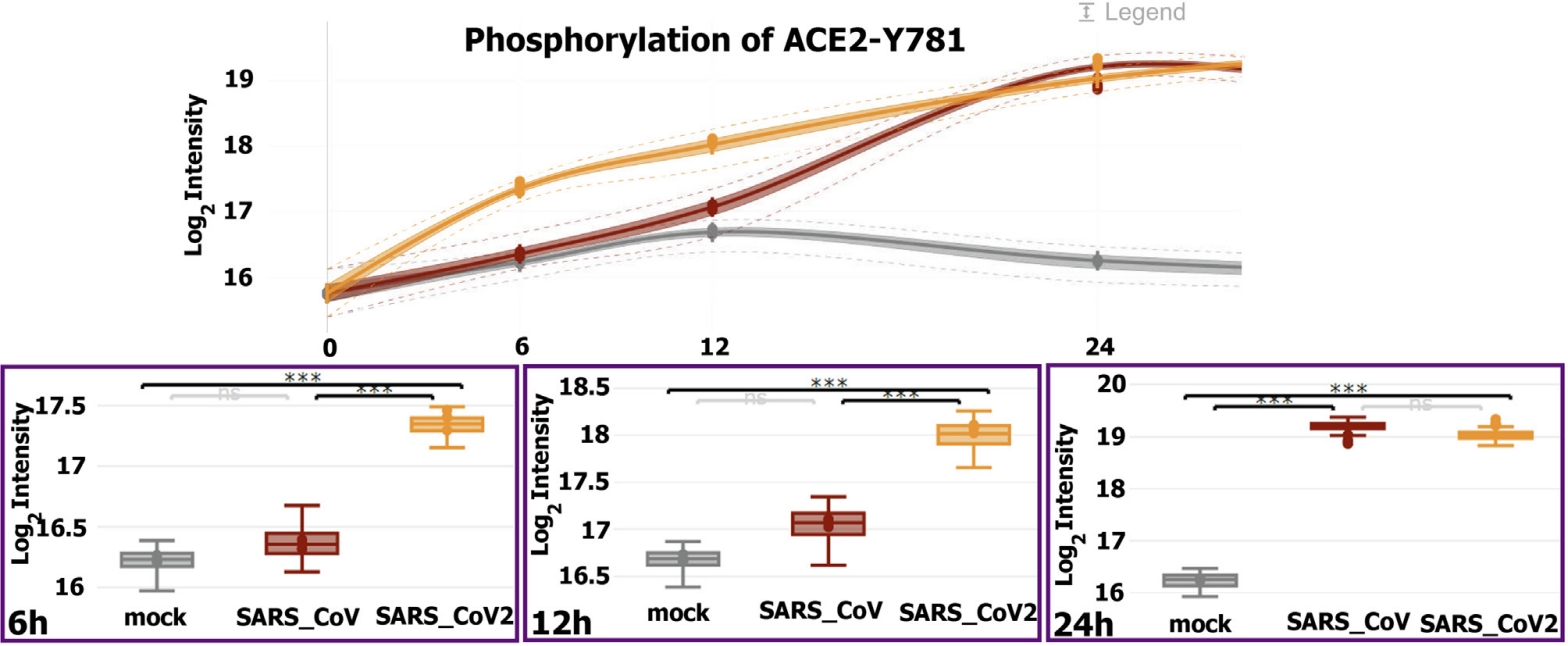
Intensity of phosphorylation of ACE2-Y781 in mock, SARS-COV, and SARS-CoV2 after 6, 12, and 24 h.

**Fig. 4:**
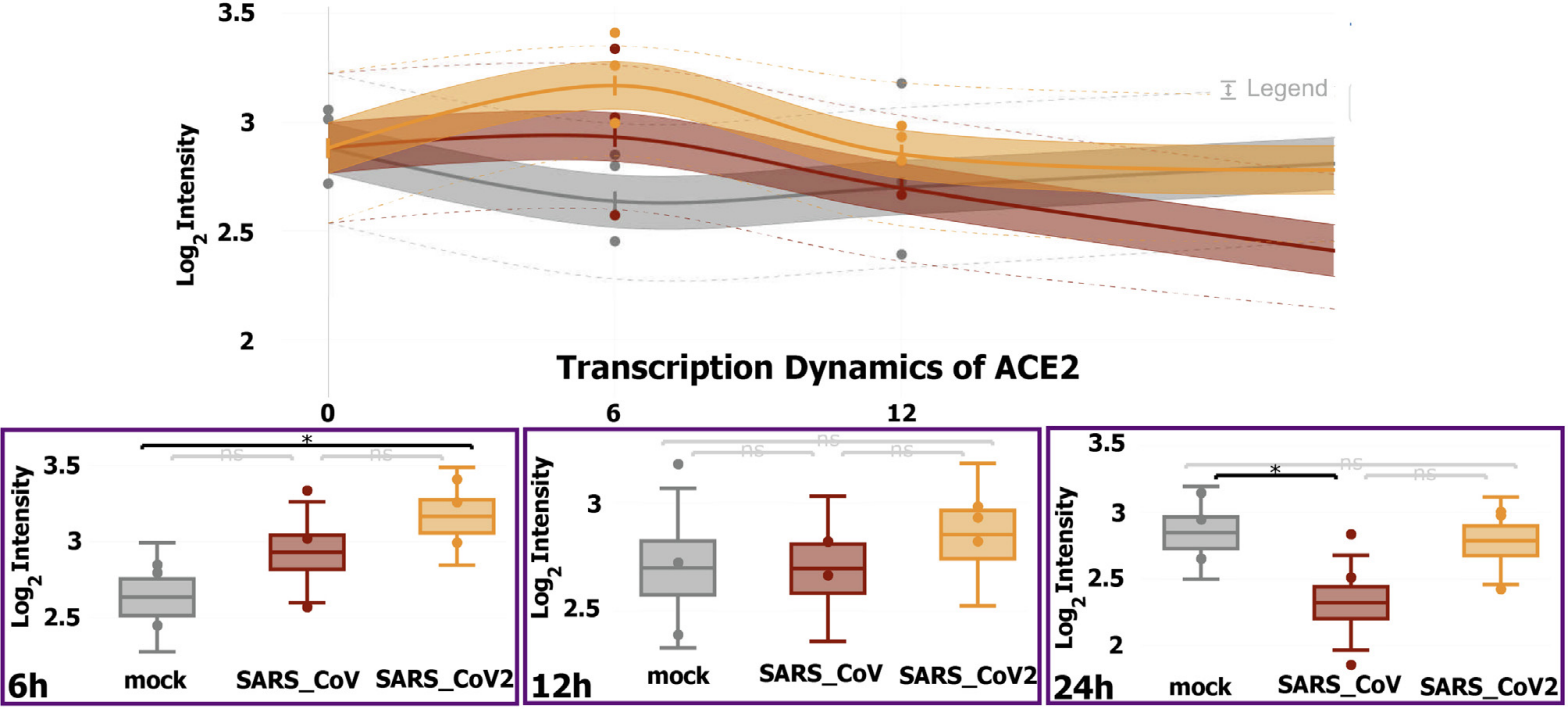
Transcription dynamic of ACE2 in mock, SARS-COV, and SARS-CoV2 after 6, 12, and 24 h.

**Fig. 5:**
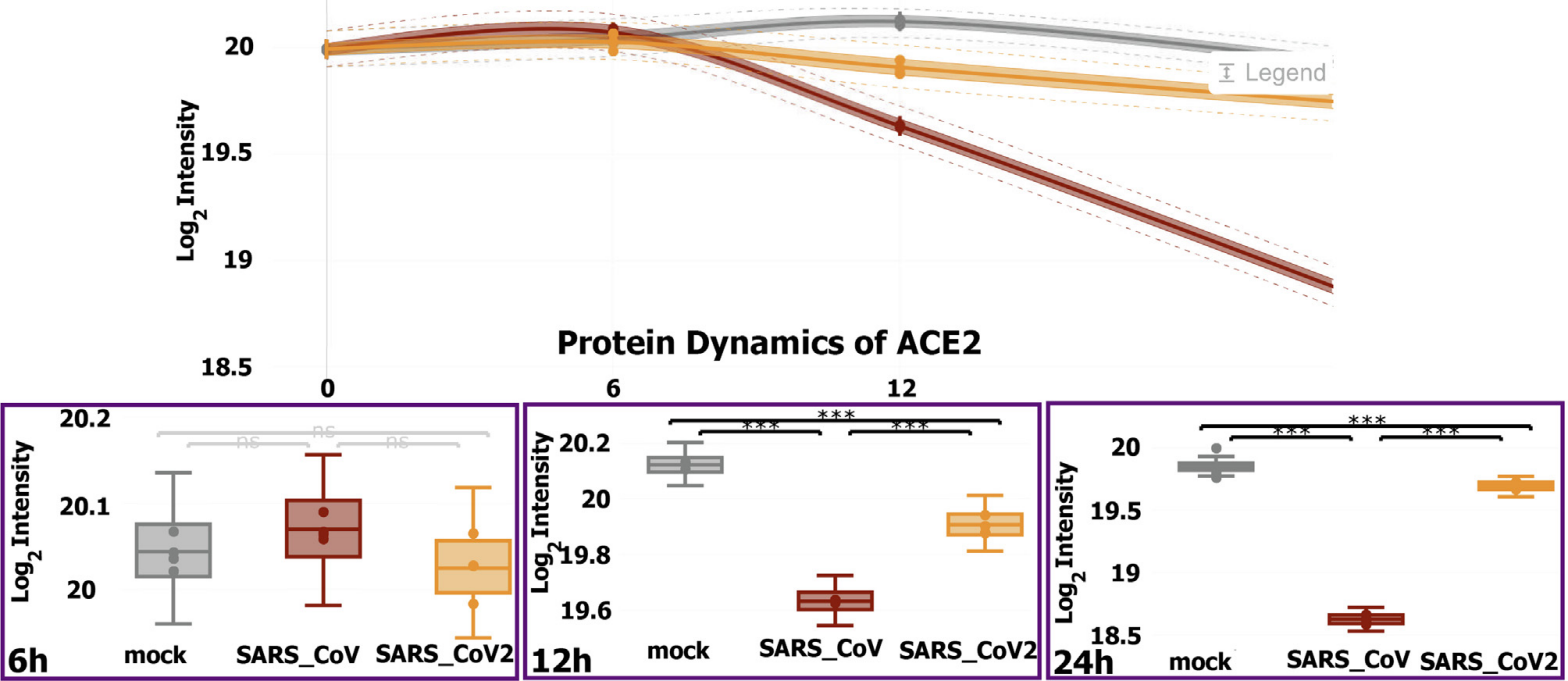
Protein dynamic of ACE2 in mock, SARS-COV, and SARS-CoV2 after 6, 12, and 24 h.

Using the COVINET database, we discovered that SARS-CoV2 induces phosphorylation of many genes at distinct locations and that this phosphorylation is time-dependent after infection (6, 12, 24, and 36 h) **(**Fig. 1**)**. Substantially, as compared to SARS-CoV and mock, SARS-CoV2 produces significant time-dependent phosphorylation of ACE2 at position s787 **(**Fig. 2**)** and y781 **(**Fig. 3**).** However, our data did not detect any phosphorylation at s680. Following that, we looked at the transcription and protein dynamics of ACE2 following SARS-CoV2 infection (6, 12, and 24 h) compared with mock and SARS-CoV. Notably, SARS-CoV2 infection induces considerable transcription dynamics after 6 h but not after 12 or 24 h compared to SARS-CoV and mock infections **(**Fig. 4**)**. In contrast, SARS-CoV2 infection causes ACE2 protein dynamic after 12 and 24 h but not after 6 h **(**Fig. 5**)**.

### 3.2 SARS-CoV2 have different variants

**Fig. 6:**
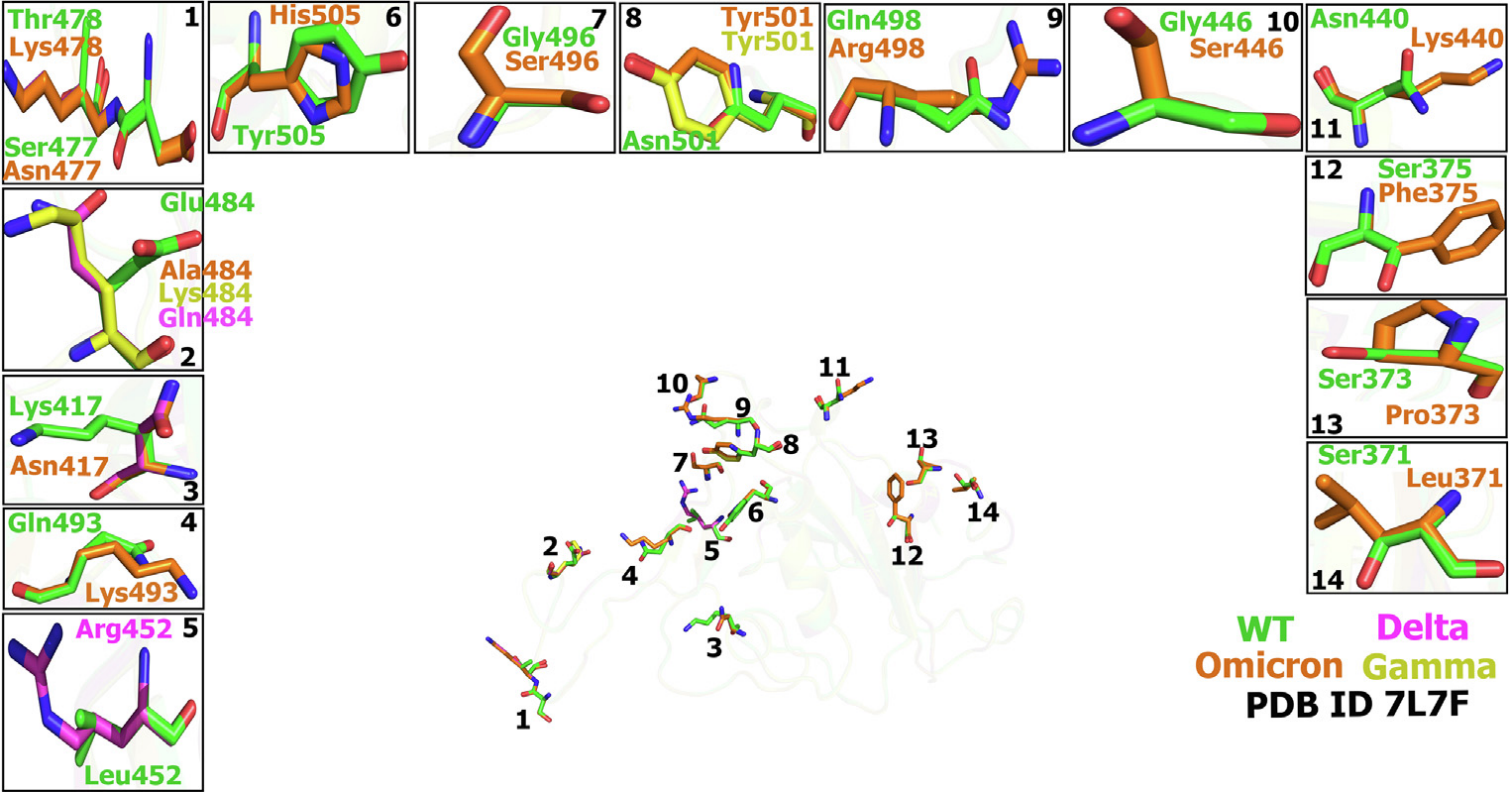
Location of the most common mutations in SARS-CoV-2 S proteins: WT, delta, omicron, and γ.

To the best of our knowledge, this is the first comprehensive molecular dynamic simulation to look at the effect of metformin-induced ACE2 phosphorylation at s680 on the interaction with several types of COVID-19 mutations compared to WT. Hence, our study selected and reported three forms of mutations including δ, omicron and γ. The δ (B.1.617.2) originated in India in October 2020. The γ (P.1) originated in Brazil/Japan in January 2021. Omicron (B.11.529) appears in multiple countries on November 2021. These variants appear to be more contagious and infectious than WT. Most repeated mutations that have been characterized in the δ variant include Gln484, and Arg452; While, γ variants include Lys484 and Tyr501**(**Fig. 6**)**. In contrast, omicron variants include Lys478, Asn477, Ala484, Asn417, Lys493, His505, Ser496, Tyr501, Arg498, Ser446, Lys440, Phe375, Pro373 and Leu371**(**Fig. 6**)**.

### 3.3 Root-mean-square deviation of atomic positions (RMSD) of WT and phosphorylated mutations

**Fig. 7:**
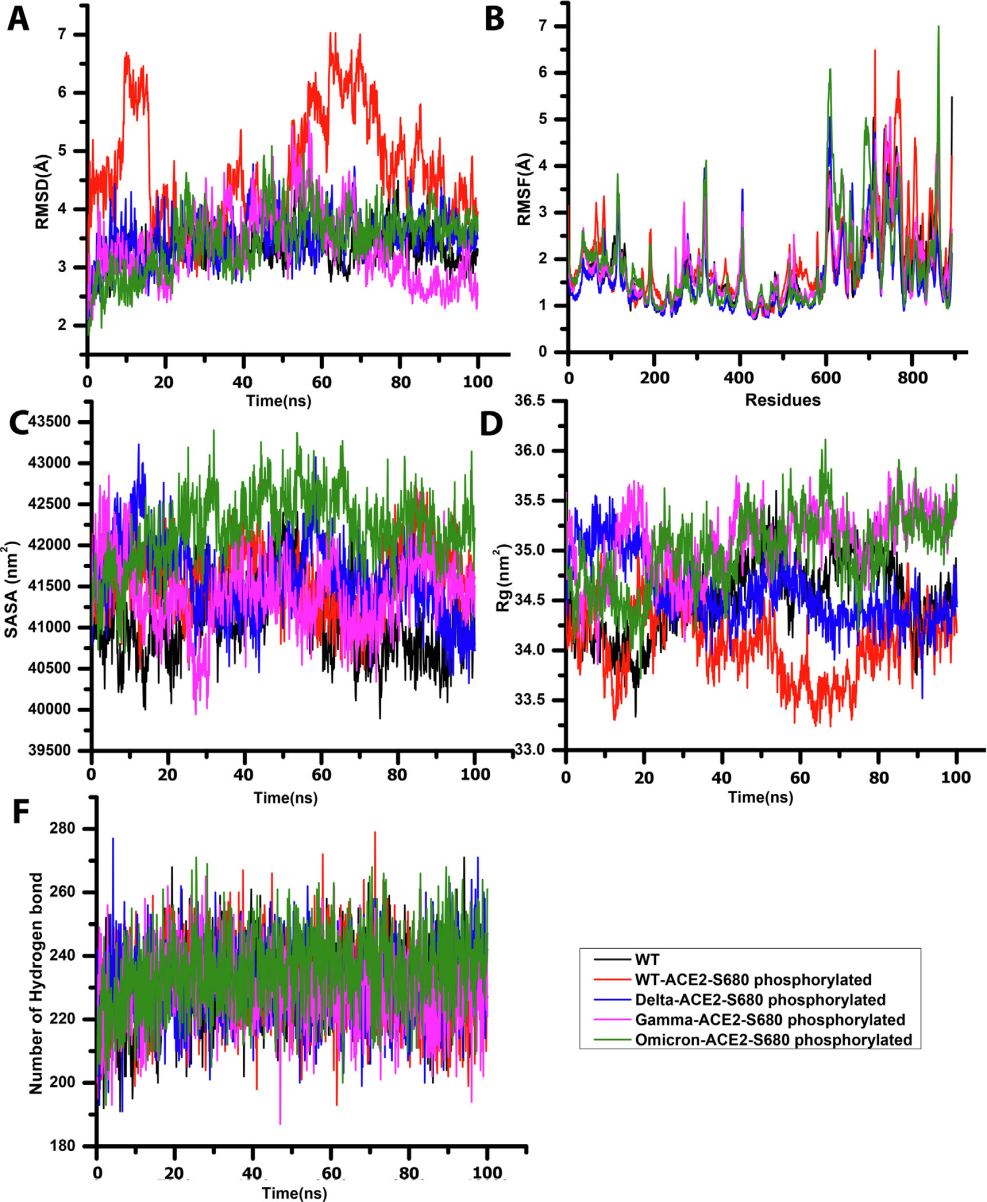
Comparison of the RMSD, RMSF, SASA, Rg, and number of HBs of WT, WT-ACE2-S680 phosphorylated, Delta-ACE2-S680 phosphorylated, γ -ACE2-S680 phosphorylated, and Omicron-ACE2-S680 phosphorylated. The results were calculated by Desmond and VMD.

**Fig. 8:**
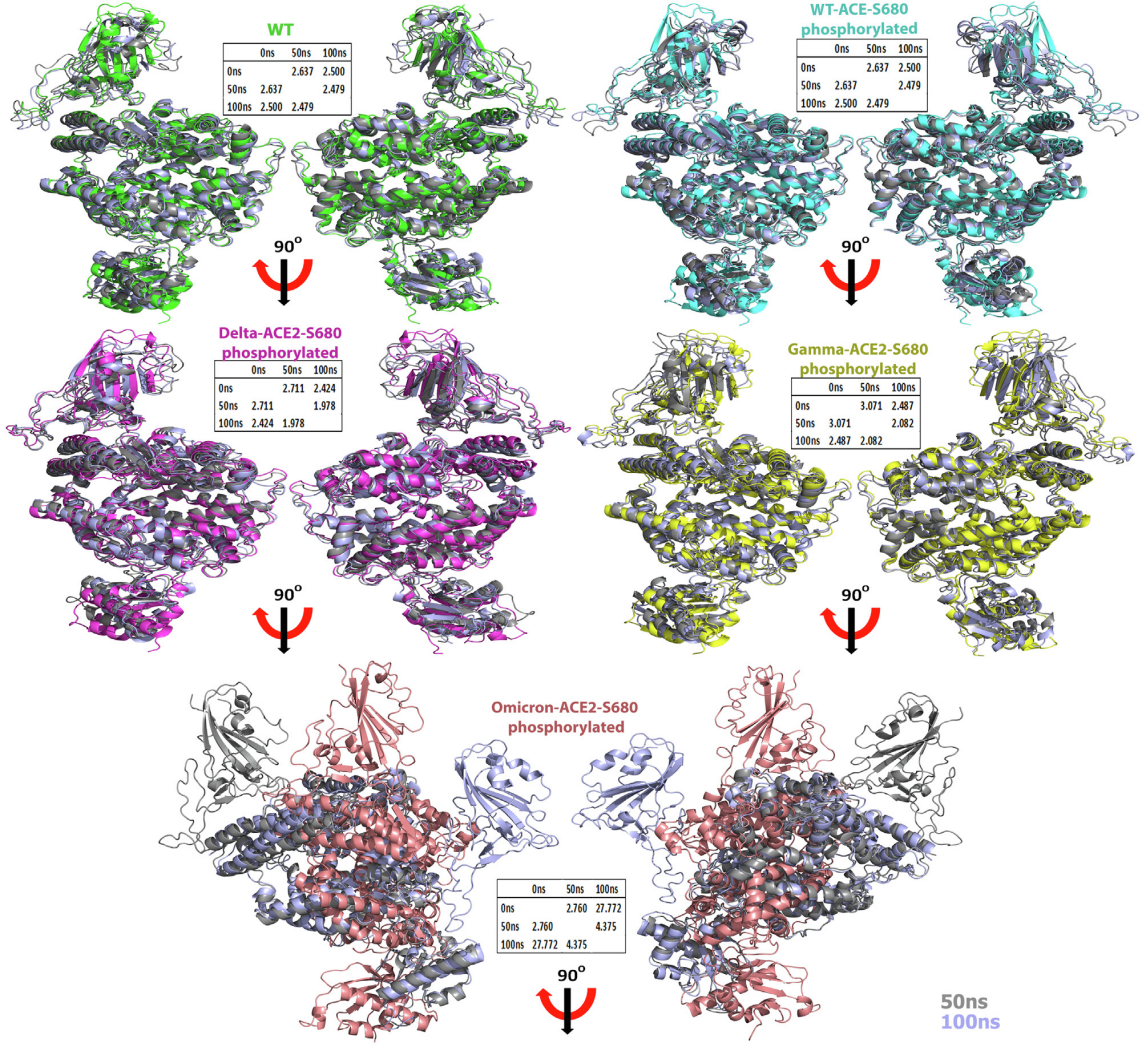
RMSD structure superimposed at 0, 25, and 50 ns simulation periods of WT, WT-ACE2-S680 phosphorylated, Delta-ACE2-S680 phosphorylated, γ-ACE2-S680 phosphorylated, and Omicron-ACE2-S680 phosphorylated. PyMOL calculated the RMSD. The cycle refers to RBD (the area where the virus binds to human protein).

The RMSD has always been used to calculate the difference between two protein structures during simulation periods by measuring the distance between the protein atoms chains. A high RMSD number denotes dissimilarity, while a zero value denotes the same conformation structure(Reva et al., 1998). The RMSD of WT against WT-ACE2-s680 phosphorylated, δ-ACE2-s680 phosphorylated, γ-ACE2-s680 phosphorylated, and omicron- ACE2-s680 phosphorylated are shown in **(**Fig. 7 **A)**. Substantially, the RMSD of WT of ACE2 without phosphorylation as control, WT-ACE2-S680-Phosphorylated, δ-ACE2-S680-Phosphorylated, γ-ACE2-S680-Phosphorylated and omicron-ACE2-S680-Phosphorylated show that among all WT and γ -ACE2-S680-Phosphorylated show high RMSD value according to Desmond and VMD **(**Fig. 7**A).** However, at the end of the simulation, the WT-ACE2-S680-Phosphorylated get increased and γ-ACE2-S680-Phosphorylated get the decrease. On the other side, when we use PyMOL to measure the RMSD, PyMOL show that omicron-ACE2-S680-Phosphorylated has a high RMSD value compared to all variant even more than the WT-ACE2-S680-Phosphorylated **(**Fig. 7**A),** while the RMSD of WT, δ-ACE2-S680-Phosphorylated get close value during all the simulation period and there is no big difference between the Desmond, VMD and PyMOL result **(**Fig. 8**)**.

### 3.4 Root mean square fluctuation (RMSF) of WT and phosphorylated mutations

The root mean square fluctuation (RMSF) method is used to calculate the flexibility of each residue and how much the residue moves or varies over the course of a simulation. In practice, structure refers to which amino acids in the protein sequence cause greater structural motion. Previous research has found that the RMSF is connected to protein function(Berhanu and Masunov, 2011, Bavi et al., 2016). First is important here to say that from 1 to 714 is ACE2 while from 715 to 897 is Virus S protein. In general, the amino acid of the virus protein gets a high RMSF value compared to the ACE2, which means the phosphorylation or any change that occurred in the ACE2 can refract on the virus S protein **(**Fig. 7**B)**. The RMSF result show virus protein can refold to be adept with the change occurring in human ACE2, which agrees with our previous finding when we run the MD of S proteins of SARS-CoV-2 variants (WT, South African, United Kingdom, Californian, Brazil) whole protein sequence individually without ACE2(Abdalla et al., 2022). According to the previous report, the N501Y mutation variant had resistant to some vaccine. As we know, omicron and γ variants have this mutation(Abdalla et al., 2022).

### 3.5 Solvent-accessible surface area (SASA) of WT and phosphorylated mutations

Solvent-accessible surface area (SASA) is used to measure the surface area of proteins accessible to a solvent(Bogatyreva and Ivankov, 2008, Shaytan et al., 2009). Our results show variation in SASA value between our samples, as omicron-ACE2-S680-Phosphorylated has a high value of SASA, WT has lost value, and lost SASA value indicates to the small surface can bind to the human protein **(**Fig. 7**C)**. In the end simulation range of SASA value for all variants was confined between 40,500 to 43000 nm^2^.

### 3.6 Radius of gyration (Rg) of WT and phosphorylated mutations

Radius of gyration (Rg) is the RMS distance between all electrons and their center of gravity, which is used to calculate the elastic stability of a cross-section(Lobanov et al., 2008). As a result of the energy interaction and the conformational entropy between residues, a low Rg value suggests tight packing. Among all samples of variant proteins, WT-ACE2-S680-Phosphorylated has lost the value of Rg because some α-helix has been decomposition during the simulation. Loss Rg value reflecting the lowest tightness packing value compared to other variants might reach the aggregating stage. During the simulation, omicron-ACE2-S680-Phosphorylated and γ-ACE2-S680-Phosphorylated get a high value of Rg compared to another variant, meaning both might have a close folding state compared with another variant. While in terms of folding stability, δ-ACE2-S680-Phosphorylated might has a better folding stability state compared with other variant because it gets stable from 25 ns up to the end of simulation. Increasing the folding will allow more surface accessibility for solvent or drugs. As RMSD or RMSF or Rg is an indicator of changing conformation inside the protein, any change in this indicator will affect the function and activity.

### 3.7 Number of hydrogen bonds (HB) and protein secondary structure elements (SSE) of WT and phosphorylated mutants

**Fig. 9:**
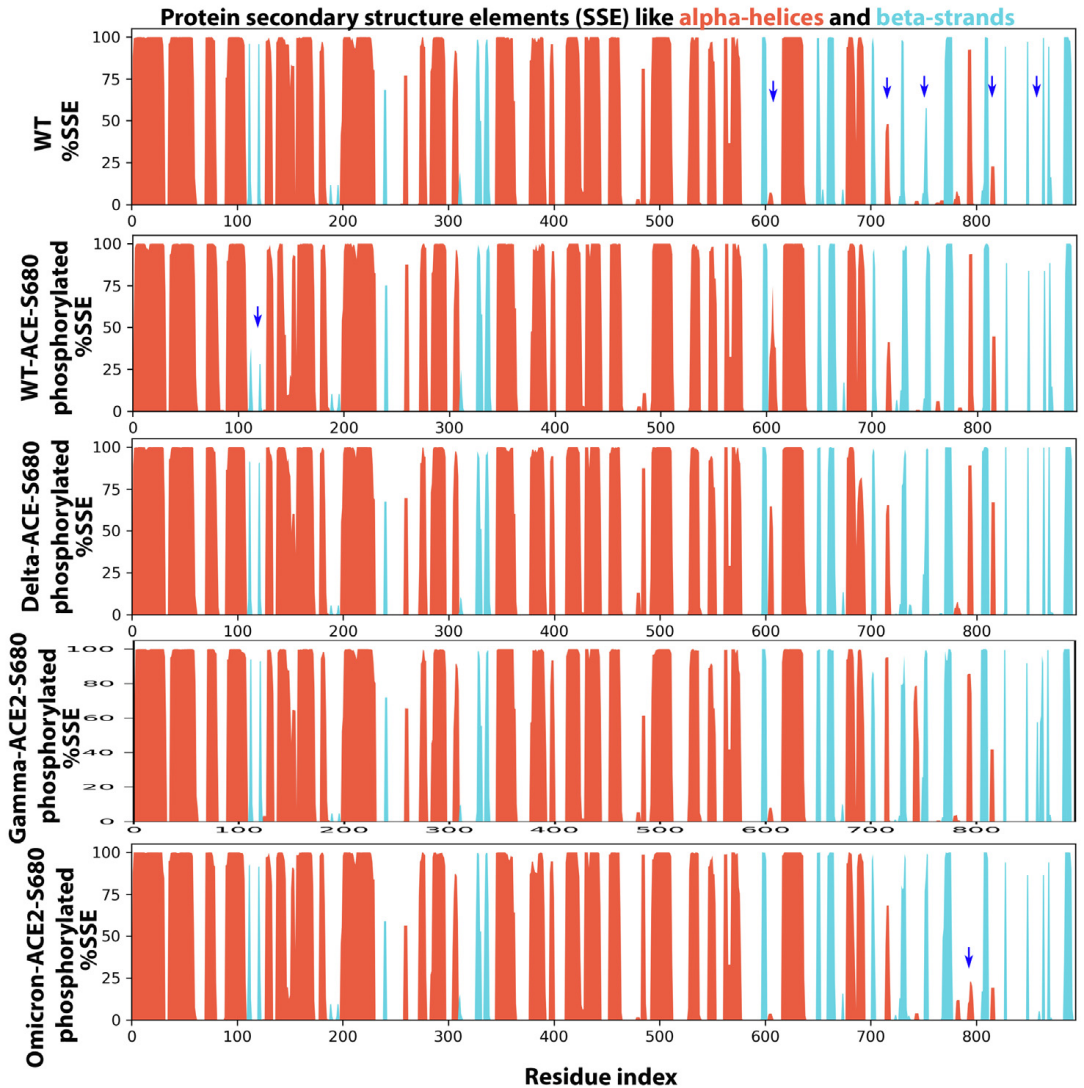
Changes in the residue index in the formation of protein SSE, such as alpha-helices and beta-strands, are characterized during the simulation times of WT, WT-ACE2-S680 phosphorylated, Delta-ACE2-S680 phosphorylated, γ-ACE2-S680 phosphorylated and Omicron-ACE2-S680 phosphorylated of SARS-CoV-2. The blue arrow indicates the difference. (For interpretation of the references to color in this figure legend, the reader is referred to the Web version of this article.).

Protein folding, stability, and flexibility are influenced by HB, hydrophobic, ionic, van der Waals, electrostatic forces, and water bridges. The HB is the most significant of them because it permits the binding of the to reside to create the 3D structure in shape and the hydrogen link occurs mostly between the carbonyl oxygen and the amide nitrogen in the protein chain(Shaytan et al., 2009, Abdalla et al., 2022). The HB, is involved in protein folding as well as stability. Among them, HB is necessary to form the 3D structure, the omicron-ACE2-S680-Phosphorylated has a high number of HB while the γ-ACE2-S680-Phosphorylated has less amount of HB **(**Fig. 7**F).** SSE is used to calculate the changes in the protein's 3D structure that occur during the simulation period for each frame in the simulation. The SSE index indicates the fraction of alpha-helices (α) and beta-strands (β) that occur per residue throughout the simulation period(Abdalla et al., 2022). The SSE is a 3D structure change indicator during 100 ns, showing the percentage α and β in the protein sequence, WT has less amounts of β compared with other variants Figure **(**Fig. 9 **and Supplementary 1) (**Table 1**).**

**Table 1:** Percentage of α Helix and β Strand in the 3D structure during the simulation.

Variants	% Helix	% Strand	% Total SSE
WT	41.25	7.58	48.83
WT-ACE-S680 phosphorylated	41.04	8.06	49.10
δ-ACE2-S680 phosphorylated	41.74	8.23	49.96
γ -ACE2-S680 phosphorylated	41.44	8.37	49.81
Omicron-ACE2-S680 phosphorylated	40.31	8.04	48.35

### 3.8 Study the conformational changes on the regulation of the ACE2-B^0^AT1 complex

ACE2 has been identified as a molecular chaperone of sodium-dependent transporters, including the major neutral amino acid (B^0^AT1) in the small intestine. The ACE2-B^0^AT1 complex was shown to be important in immunoregulation via regulating amino acid homeostasis, antimicrobial peptide expression, and the ecological modulation of gut bacteria(Zhang et al., 2022). It is generally known that three key locations on ACE2 (K625, R678, and L760) interact with B0AT1 to produce HB. As a result, we investigated the effect of ACE2-S680-Phosphorylated on these critical locations(Loo and Wright 2021). Since the RMSF results through the Desmond and VMD did not show a clear difference, we tried to zoom inside the MD structure using PyMole to know the amount of change and shifting in the space as we chose Lys625, Lys676 and Arg678 as we tried to present in 3-time points 0, 50 and 100 ns, the Result show that in case of Lys625 omicron-ACE2-S680-Phosphorylated has a high amount of shift or fluctuations 29 Å between 0 and 100 ns, then δ-ACE2-S680-Phosphorylated with 11.6 Å between 0 and 100 ns, while WT-ACE2-S680-Phosphorylated is 7.4 Å. In the case of lys676 and Arg 678, the shift amount is less than in Lys625 **(Supplementary 2 &3 & 4).**

## 4 Discussion

Since the discovery of COVID-19 (SARSCoV2) in Wuhan, Hubei Province, China in December 2019, healthcare systems worldwide have faced enormous hurdles(El-Arabey and Abdalla 2020). In this regard, with the ongoing COVID-19 pandemic, it is critical to developing drugs that enhance COVID-19 outcomes. Metformin, an oral antihyperglycemic, appears to be related to lower COVID-19 severity in people with diabetes as compared to other antihyperglycemic medicines. Some individuals without diabetes, such as those with the polycystic ovarian syndrome, obesity and prediabetes, are administered metformin for off-label usage, providing an opportunity to examine further metformin's influence on COVID-19(El-Arabey, 2018, El-Arabey and Abdalla, 2020). A recent prediabetes cohort research indicated that metformin was associated with less severe COVID-19 in individuals with prediabetes, as seen in previous diabetic studies(Ma et al., 2020). Therefore, the present study aimed to investigate the interaction of the phosphorylated form of ACE2-s680 WT and/ or other SARS-CoV-2 infection mutations on host cell entry, as well as the regulation of B0AT1 by the SARS-CoV-2 receptor ACE2.

The SARS-CoV-2 infection activates host kinases and causes heavy phosphorylation in both the host and the virus. Notably, the virus was phosphorylated using the host enzyme for better survival and further mutations. Besides, SARS-CoV2 induces phosphorylation of many genes at distinct locations, and this phosphorylation is time-dependent after infection(Chatterjee and Thakur, 2022, Mohapatra et al., 2022). Herein, we illustrated that SARS-CoV2 produces significant time-dependent phosphorylation of ACE2 at position s787 and y781 but not at s680 when compared with SARS-CoV and mock. As a result, metformin's phosphorylation of ACE2 at s680 appears to be useful for treatment purposes. Angiotensin-converting enzyme-2 is a key component of the renin-angiotensin system (RAS) axis and a key entry point for SARS-CoV-2, the dynamics of ACE2 expression in response to various extrinsic and intrinsic factors are of current interest. Therefore, understanding the ACE2 dynamic as a critical interaction between the covid-19 pandemic and other communicable and non-communicable diseases is an attractive approach(Rath et al., 2021). In this regard, our analysis demonstrated that SARS-CoV2 infection induces significant transcription dynamics only after 6 h. On the other hand, SARS-CoV2 infection causes ACE2 protein dynamic after 12 and 24 h but not after 6 h. SARS-CoV-2 particles reach respiratory ciliated cells after inhalation via interacting with ACE2. This explains why SARS-CoV2 infection generates considerable ACE2 transcription dynamics after 6 h, followed by protein dynamics after 12 and 24 h(Manna et al., 2022). COVID-19 can cause gastrointestinal (GI) side effects in addition to the typical systemic (fever) and respiratory (cough, dyspnea) symptoms(Ma et al., 2020). GI symptoms have been described in 11.4–61.1% of COVID-19 patients, with varying onset and severity. Anorexia, diarrhea, nausea, vomiting, and stomach pain/discomfort are the most common COVID-19-related GI symptoms. A small percentage of patients have an acute abdomen caused by an underlying condition such as acute pancreatitis, acute appendicitis, intestinal obstruction, bowel ischemia, hemoperitoneum, or abdominal compartment syndrome. Patients with GI symptoms also had a longer time between the beginning of symptoms and viral clearance, and they were more likely to have a virus-positive stool test(Bilal et al., 2021, Kariyawasam et al., 2021, Pola et al., 2021). In the active phase of COVID-19, pulmonary symptoms are the hallmark of severe cases. At the same time, approximately half of the COVID-19 patients exhibit extrapulmonary GI tropism with gut clinical symptomology accompanied by virion particles shed in feces and RNA in toilet aerosols, and intestinal symptoms persist in long-term post-acute sequelae of SARS-CoV-2 (PASC)(Stevens et al., 2021, Almutairi et al., 2022). According to the Shanghai experience, gastrointestinal symptoms caused by Omicron variations are rather infrequent compared to other variants. The researchers observed that the Omicron variant strain had a lower proliferation efficiency in the intestinal model(Shi et al., 2022).

This is the first research to highlight metformin's potential function in modulating GI adverse effects via the ACE2-B^0^AT1 complex. These elements combine to form a [ACE2:B^0^AT1]2 dimer-of-heterodimer quaternary complex that is thought to direct SARS-CoV-2 tropism in the GI tract. GI clinical symptomology has been recorded in around half of COVID-19 patients, and it can be accompanied with virion particle shedding in the gut. Besides, in local gut mucosa renin–angiotensin system regulating absorption of sodium and organic nutrients(Stevens et al., 2021).

## 5 Conclusions

Here, we presented the stability and flexibility changes of phosphorylated-ACE2-S680 in WT and variants of SARS-CoV-2 by using MD simulation. The homologous region between the phosphorylated-ACE2-S680 forms of WT and variants is high in S protein when compared with WT. However, the interaction patterns showed variation with different degrees of flexibility to induce their effect **(Supplementary 5)**. Our findings revealed a considerable shift in all SARS-CoV-2 variants with the critical sites of the ACE2-B^0^AT1 complex, with the largest fluctuations occurring in the case of omicron-ACE2-S680-Phosphorylated.These findings contribute to our understanding of metformin's potential mechanism, which will aid in future translational research aimed at treating or mitigating COVID-19 variant breakouts and/or GI symptoms persistence in long-term PASC.

## 6 Authors' contributions

A.A.E and M.A wrote the main manuscript and run the MD, A.R, A.A.E, H.S.M, Y.A.M, A.S.A, prepared figures, All authors read and approved the final manuscript.

## Funding

The authors extend their appreciation to the Deputyship for Research & Innovation, Ministry of Education in Saudi Arabia for funding this research work through the project number RI-44–0251.

## Declaration of Competing Interest

The authors declare that they have no known competing financial interests or personal relationships that could have appeared to influence the work reported in this paper.

## Figures and Tables

**Supplementary figure 1:**
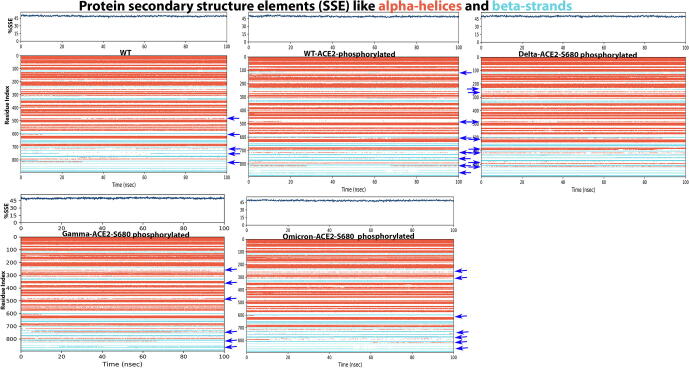


**Supplementary figure 2:**
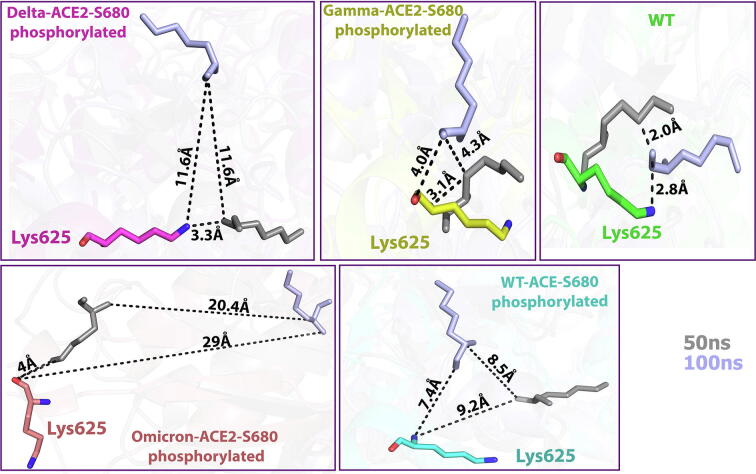


**Supplementary figure 3:**
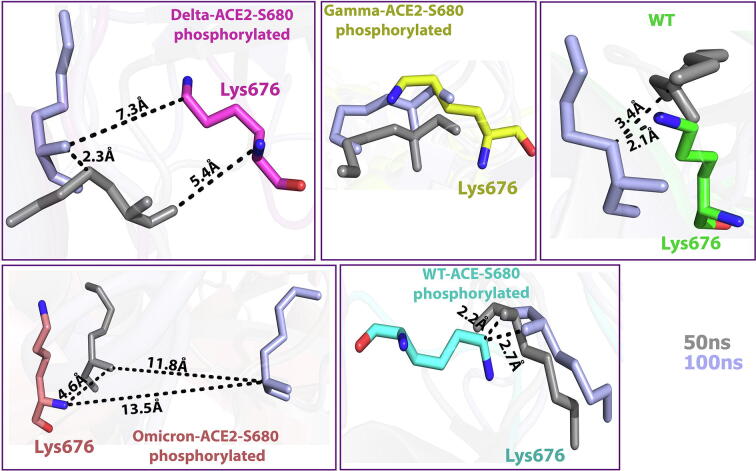


**Supplementary figure 4:**
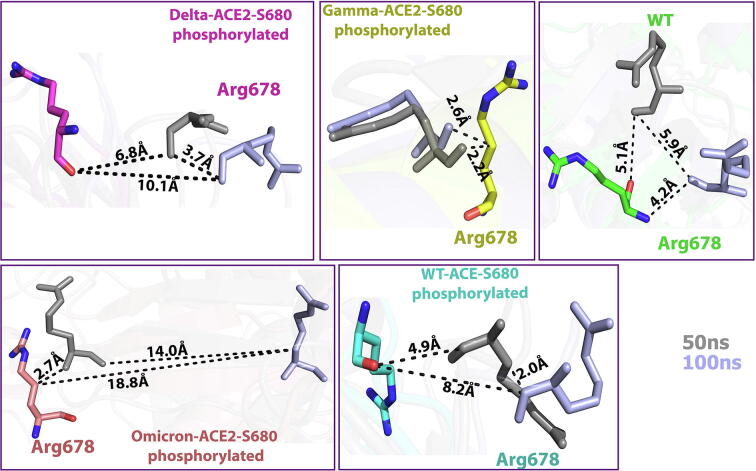


**Supplementary figure 5:**