# Supplementary material for: Molecular dynamic and bioinformatic studies of metformin-induced ACE2 phosphorylation in the presence of different SARS-CoV-2 S protein mutations
Source: Saudi J Biol Sci. 2023 Jun 3;30(7):103699. doi: 10.1016/j.sjbs.2023.103699 (PMC10238124; doi:10.1016/j.sjbs.2023.103699)
Supplement: Supplementary data 1 [file mmc1.pdf]

Molecular dynamic and bioinformatic studies of metformin-induced ACE2 phosphorylation in the presence of different SARS-CoV-2 S protein-mutations

by Mohanad Abdalla

Submission date: 20-Apr-2023 10:15AM (UTC+0800)
Submission ID: 2069898066
File name: MS_metformin_-_Copy_2_-_Copy.docx (98.18K)
Word count: 3166
Character count: 22120

Molecular dynamic and bioinformatic studies of metformin-induced ACE2 phosphorylation in the presence of different SARS-CoV-2 S protein mutations

Abstract:

The SARS-CoV-2 infection activates host kinases and causes high phosphorylation in both the virus and host. There were around seventy phosphorylation sites found in SARS-CoV-2 viral proteins. Besides, almost fifteen thousand-host phosphorylation sites were found in SARS-CoV-2-infecte cells. COVID-19 is thought to enter cells via the well known receptor the serine protease TMPRSS2 as well as Angiotensin Converting Enzyme 2 (ACE2). Substantially, the COVID-19 infection doesn't induce phosphorylation of the ACE2 receptor at Serin-680(s680). Metformin's numerous pleiotropic properties and extensive use in medicine including COVID-19, have inspired experts to call it the "aspirin of the twenty-first century". Metformin's impact on COVID-19 has been verified in clinical investigations via ACE2 receptor phosphorylation at s680. In the infection of COVID-19, sodium dependent transporters including the major neutral amino acid (B^0AT1) is regulated by ACE2. The structure of B^0AT1 complexing with the COVID-19 receptor ACE2 enabled significant progress in the creation of mRNA vaccines. We aimed to study the impact of the interaction of the phosphorylation form of ACE2-s680 with wild-type (WT) and different mutations of SARS-CoV-2 infection such as delta, omicron, and gamma on their entrance of host cells as well as the regulation of $B^0AT1$by the SARS-CoV-2 receptor ACE2. Interestingly, compared to WT SARS-CoV-2, ACE2 receptor phosphorylation at s680 produces conformational alterations in all types of SARS-CoV-2. Furthermore, our results showed for the first time that this phosphorylation significantly influences ACE2 sites K625, K676, and R678, which are key mediators for ACE2-B^0AT1 complex.

Keywords: SARS-CoV-2; COVID-19; ACE2; B^0AT1; metformin; ACE2-phosphorylation at s680.

1. Introduction:

In China, a severe acute respiratory illness caused by coronavirus emerged in 2002. Ten years later, in 2012, a new strain of the Middle East Respiratory Syndrome Coronavirus virus emerged in the Middle East. The Chinese Center for Disease Control and Prevention (China CDC) reported pneumonia of unknown origin at the end of 2019. The pneumonia was connected to a wet animal and seafood wholesale market in Wuhan, Hubei Province, China, and was eventually identified as COVID-19. The current worldwide coronavirus disease 2019 (COVID-19) epidemic is caused by severe acute respiratory syndrome coronavirus 2. In this way, COVID-19 has presented enormous hurdles to healthcare systems all around the world since its discovery (El-Arabey and Abdalla 2020). COVID-19 has affected more than 231 million people worldwide and caused 6,544,762 deaths in 200 countries as of September 30 2022, https://coronavirus.jhu.edu/map.

Several investigations have concluded that COVID-19 enters cells via the well known receptor Angiotensin Converting Enzyme 2 (ACE2) and the serine protease TMPRSS2. The interaction of ACE2 with the SARS-CoV-2 spike protein is essential for viral entry and subsequent infection (Pique-Regi et al., 2020, El-Arabey and Abdalla 2021). ACE2 is largely expressed by epithelial cells of the lung ,stomach, kidney, and blood vessels. This might explain why persons with severe COVID-19 infections are more likely to develop pneumonia and bronchitis. According to a recent study, ACE2 is also highly expressed in the mucosa of the oral cavity, allowing the virus to infect a new susceptible host (Ciaglia et al., 2020). As a result, blocking this binding event or reducing the virus's accessibility to the ACE2 receptor is an alternative technique for avoiding COVID-19. Furthermore, ACE2's biological significance in regulating the innate immune system and tissue repair pathways underlines its therapeutic potential in treating infected persons (Jia et al., 2021). Preclinical data shows that after SARS-CoV-2 binding, ACE2 may be downregulated, and treatments that increase ACE2 may protect against cardiopulmonary injury (Malhotra et al., 2020). According to recent research, monitoring plasma ACE2 may be effective in predicting COVID-19 outcomes. Furthermore, ACE2 is linked to the severity of COVID-19 illness and known risk factors such as hypertension, pre-existing heart disease, and pre-existing renal disease (Kragstrup et al., 2021).

It suggests that ACE2 is involved in the link between diabetes and COVID-19. In people with diabetes, ACE2 expression is reduced, most likely due to glycosylation; this might explain the increased risk of severe lung injury and respiratory distress syndrome with COVID-19(Pal and Bhansali 2020).

Metformin was found in the 1940s while looking for antimalarial drugs and proved beneficial in treating influenza during a clinical trial by reducing blood glucose. Metformin was licensed by the Food and Drug Administration (FDA) in 1995 as an oral hypoglycemic agent, and it has since become one of the most commonly prescribed diabetes medications in the world, with the potential for further therapeutic applications(El-Arabey and Abdalla 2020). Several studies in recent years have highlighted metformin's potential usefulness as a promising medicine for treating polycystic ovarian syndrome, cancer , aging, cardiovascular disease, metabolic syndrome, and neurological illnesses. Furthermore, it is used off label for weight loss in the United States (Wang et al., 2016, El-Arabey 2018). Metformin appears to have unique activities in treating autoimmune illness, including decreased macrophage cytokine production. Furthermore, it has been proposed that metformin may suppress the virus by improving insulin sensitivity (El-Arabey and Abdalla 2020). According to many observational retrospective investigations, metformin users had a lower mortality rate than non users. Metformin's impact may be attributed to numerous mechanisms, including improved glucose regulation, increased cellular pH, a decrease of (body weight, insulin resistance, neutrophils, mitochondrial reactive oxygen species, inflammatory mediators), and blockage of the mTOR pathway(Scheen 2020). Furthermore, metformin acts as an AMPK activator, causing ACE2 phosphorylation at s680(Varghese et al., 2021). Besides, it was proposed theoretically that metformin may decrease the penetration of SARS-CoV-2 to the host cells(Scheen 2020).

The ACE2 enhances sodium-dependent transporters, including the major neutral amino acid (B^0AT1) trafficking to the plasma membrane and modulates transporter activity in the oocyte expression system (Loo and Wright 2021). Aside from ACE2's role as the SARS-CoV-2 receptor and the necessity of co-expressing B^0AT1 with ACE2 in creating a COVID-19 mRNA vaccine, the 3D atomic coordinates prompted intriguing issues concerning ACE2 and B^0AT1's functional connection. Such inquiries include how ACE2 regulates B^0AT1 brush

border expression and activity in the intestine (Loo and Wright 2021). Based on the prior findings, there is reason to believe that metformin is a viable adjuvant therapy with routine hospital care in hospitalized patients with Severe Acute Respiratory Syndrome related to SARS-CoV2 with or without diabetes. Hence, the purpose of this study is to assess the molecular dynamic effect of ACE2-s680 phosphorylation by metformin on the interaction with wild-type (WT) and different forms of COVID-19 mutations such as Delta (δ), omicron and gamma (γ) as well as the SARS-CoV-2 receptor ACE2's control of B^0AT1.

2. Methodology

2.1. MD simulation

The PDB file 7DF4(Wang et al., 2021) was used as WT SARS-CoV-2 S protein. The MD simulation was run on Desmon with default protocols(Bowers et al., 2006). The TIP3P model in Desmond System Builder tool was used to solvate the protein. Periodic boundary conditions with 10 Å orthorhombic box were used on the outer protein surface, and 0.15 M NaCl was usedto neutralize the simulation system. The simulation was run at temperature of 310 K and pressure of 1.013 bar for 100 ns. The trajectory was analyzed by Desmond, VMD(Humphrey et al., 1996), and PyMOL.

2.2. Bioinformatics:

Using cutting-edge high-throughput omics methods, we investigated COVID-19 infection from a variety of viewpoints linked to phosphorylation and ACE2. The COVINET database includes basic knowledge of SARS-CoV2, as well as aiding in the development of antiviral drugs(Stukalov et al., 2021).

3. Results and discussion:

3.1. SARS-CoV2 induces phosphorylation at numerous genes including ACE2, which varies over time:

Using the COVINET database, we discovered that SARS-CoV2 induces phosphorylation of many genes at distinct locations and that this phosphorylation is

time-dependent after infection (6, 12, 24, and 36 hours) **(Figure 1)**. Substantially, as compared to SARS-CoV and mock, SARS-CoV2 produces significant time-dependent phosphorylation of ACE2 at position s787 **(Figure 2)** and y781 **(Figure 3).** However, our data did not detect any phosphorylation at s680. Following that, we looked at the transcription and protein dynamics of ACE2 following SARS-CoV2 infection (6, 12, and 24 hours) compared with mock and SARS-CoV. Notably, SARS-CoV2 infection induces considerable transcription dynamics after 6 hours but not after 12 or 24 hours compared to SARS-CoV and mock infections **(Figure 4)**. In contrast, SARS-CoV2 infection causes ACE2 protein dynamic after 12 and 24 hours but not after 6 hours **(Figure 5)**.

3.2. SARS-CoV2 have different variants:

To the best of our knowledge, this is the first comprehensive molecular dynamic simulation to look at the effect of metformin-induced ACE2 phosphorylation at s680 on the interaction with several types of COVID-19 mutations compared to WT. Hence, our study selected and reported three forms of mutations including δ, omicron and γ. The δ (B.1.617.2) originated in India in October, 2020. The γ (P.1) originated in Brazil/Japan in January, 2021. Omicron (B.11.529) appears in multiple countries on November 2021. These variants appear to be more contagious and infectious than WT. Most repeated mutations that have been characterized in the δ variant include Gln484, and Arg452; While, γ variants include Lys484 and Tyr501**(Figure 6)**. In contrast, omicron variants include Lys478, Asn477, Ala484, Asn417, Lys493, His505, Ser496, Tyr501, Arg498, Ser446, Lys440, Phe375, Pro373 and Leu371**(Figure 6)**.

3.3. Root-mean-square deviation of atomic positions (RMSD) of WT and Phosphorylated mutations:

The RMSD has always been used to calculate the difference between two protein structures during simulation periods by measuring the distance between the protein atoms chains. A high RMSD number denotes dissimilarity, while a zero value denotes the same conformation structure (Reva et al., 1998). The RMSD of WT against WT-ACE2-s680 phosphorylated, δ-ACE2-s680 phosphorylated, γ-ACE2-s680 phosphorylated, and omicron- ACE2-s680

phosphorylated are shown in **(Figure 7 A)**. Substantially, the RMSD of WT of ACE2 without phosphorylation as control, WT-ACE2-S680-Phosphorylated, δ-ACE2-S680-Phosphorylated, γ-ACE2-S680-Phosphorylated and omicron-ACE2-S680-Phosphorylated show that among all WT and γ -ACE2-S680-Phosphorylated show high RMSD value according to Desmond and VMD **(Figure 7A).** However, at the end of the simulation, the WT-ACE2-S680-Phosphorylated get increased and γ-ACE2-S680-Phosphorylated get the decrease. On the other side, when we use PyMOL to measure the RMSD, PyMOL show that omicron-ACE2-S680-Phosphorylated has a high RMSD value compared to all variant even more than the WT-ACE2-S680-Phosphorylated **(Figure 7A),** while the RMSD of WT, δ-ACE2-S680-Phosphorylated get close value during all the simulation period and there is no big difference between the Desmond, VMD and PyMOL result **(Figure 8)**.

3.4. Root mean square fluctuation (RMSF) of WT and Phosphorylated mutations:

The root mean square fluctuation (RMSF) method is used to calculate the flexibility of each residue and how much the residue moves or varies over the course of a simulation. In practice, structure refers to which amino acids in the protein sequence cause greater structura motion. Previous research has found that the RMSF is connected to protein function (Berhanu and Masunov 2011, Bavi et al., 2016). First is important here to say that from 1 to 714 is ACE2 while from 715 to 897 is Virus S protein. In general, the amino acid of the virus protein gets a high RMSF value compared to the ACE2, which means the phosphorylation or any change that occurred in the ACE2 can refract on the virus S protein **(Figure 7B)**. The RMSF result show virus protein can refold to be adept with the change occurring in human ACE2, which agrees with our previous finding when we run the MD of S proteins of SARS-CoV-2 variants (WT, South African, United Kingdom, Californian, Brazil) whole protein sequence individually without ACE2(Abdalla et al., 2022). According to the previous report, the N501Y mutation variant had resistant to some vaccine. As we know, omicron and γ variants have this mutation(Abdalla et al., 2022).

3.5. Solvent-accessible surface area (SASA) of WT and Phosphorylated mutations:

Solvent-accessible surface area (SASA) is used to measure the surface area of proteins accessible to a solvent (Bogatyreva and Ivankov 2008, Shaytan et al., 2009). Our results show variation in SASA value between our samples, as omicron-ACE2-S680-Phosphorylated has a high value of SASA, WT has lost value, and lost SASA value indicates to the small surface can bind to the human protein (**Figure 7C**). In the end simulation range of SASA value for all variants was confined between 40500 to 43000 nm^2.

3.6. Radius of gyration (Rg) of WT and Phosphorylated mutations:

Radius of gyration (Rg) is the RMS distance between all electrons and their center of gravity, which is used to calculate the elastic stability of a cross-section (Lobanov et al., 2008). As a result of the energy interaction and the conformational entropy between residues, a low Rg value suggests tight packing. Among all samples of variant proteins, WT-ACE2-S680-Phosphorylated has lost the value of Rg because some α-helix has been decomposition during the simulation. Loss Rg value reflecting the lowest tightness packing value compared to other variants might reach the aggregating stage. During the simulation, omicron-ACE2-S680-Phosphorylated and Gamma-ACE2-S680-Phosphorylated get a high value of Rg compared to another variant, meaning both might have a close folding state compared with another variant. While in terms of folding stability, δ-ACE2-S680-Phosphorylated might has a better folding stability state compared with other variant because it gets stable from 25 ns up to the end of simulation. Increasing the folding will allow more surface accessibility for solvent or drugs. As RMSD or RMSF or Rg is an indicator of changing conformation inside the protein, any change in this indicator will affect the function and activity.

3.7. Number of hydrogen bonds (HB) and Protein secondary structure elements (SSE) of WT and Phosphorylated mutants:

Protein folding, stability, and flexibility are influenced by HB, hydrophobic, ionic, van der Waals, electrostatic forces, and water bridges. The HB is the most significant of them because it permits the binding of the to reside to create the 3D structure in shape and the hydrogen link occurs mostly between the

carbonyl oxygen and the amide nitrogen in the protein chain (Shaytan et al., 2009, Abdalla et al., 2022). The HB, is involved in protein folding as well as stability. Among them, HB is necessary to form the 3D structure, the omicron-ACE2-S680-Phosphorylated has a high number of HB while the γ-ACE2-S680-Phosphorylated has less amount of HB **(Figure 7F).** SSE is used to calculate the changes in the protein's 3D structure that occur during the simulation period for each frame in the simulation. The SSE index indicates the fraction of alpha-helices (α) and beta-strands (β) that occur per residue throughout the simulation period (Abdalla et al., 2022). The SSE is a 3D structure change indicator during 100 ns, showing the percentage α and β in the protein sequence, WT has less amounts of β compared with other variants **(Figures 9 and 10) (Table 1).**

3.8. Study the conformational changes on the regulation of the ACE2-B^0AT1 complex:

ACE2 has been identified as a molecular chaperone of sodium dependent transporters, including the major neutral amino acid (B^0AT1) in the small intestine. The ACE2-B^0AT1 complex was shown to be important in immunoregulation via regulating amino acid homeostasis, antimicrobial peptide expression, and the ecological modulation of gut bacteria(Zhang et al., 2022). It is generally known that three key locations on ACE2 (K625, R678, and L760) interact with B0AT1 to produce HB. As a result, we investigated the effect of ACE2-S680-Phosphorylated on these critical locations(Loo and Wright 2021). Since the RMSF results through the Desmond and VMD did not show a clear difference, we tried to zoom inside the MD structure using PyMole to know the amount of change and shifting in the space as we chose Lys625, Lys676 and Arg678 as we tried to present in 3-time points 0, 50 and 100 ns, the Result show that in case of Lys625 omicron-ACE2-S680-Phosphorylated has a high amount of shift or fluctuations 29 Å between 0 and 100ns, then δ-ACE2-S680-Phosphorylated with 11.6 Å between 0 and 100ns, while WT-ACE2-S680-Phosphorylated is 7.4 Å. **(Figure 11)**. In the case of lys676 and Arg 678, the shift amount is less than in Lys625 **(Figure 12).**

4. Discussion:

Since the discovery of COVID-19 (SARS-CoV-2) in Wuhan, Hubei Province, China in December 2019, healthcare systems worldwide have faced enormous hurdles(El-Arabey and Abdalla 2020). In this regard, with the ongoing COVID-19 pandemic, it is critical to developing drugs that enhance COVID-19 outcomes. Metformin, an oral antihyperglycemic, appears to be related to lower COVID-19 severity in people with diabetes as compared to other antihyperglycemic medicines. Some individuals without diabetes, such as those with the polycystic ovarian syndrome, obesity and prediabetes, are administered metformin for off-label usage, providing an opportunity to examine further metformin's influence on COVID-19(El-Arabey 2018, El-Arabey and Abdalla 2020). A recent prediabetes cohort research indicated that metformin was associated with less severe COVID-19 in individuals with prediabetes, as seen in previous diabetic studies(Ma et al., 2020). Therefore, the present study aimed to investigate the interaction of the phosphorylated form of ACE2-s680 WT and/ or other SARS-CoV-2 infection mutations on host cell entry, as well as the regulation of B0AT1 by the SARS-CoV-2 receptor ACE2.

The SARS-CoV-2 infection activates host kinases and causes heavy phosphorylation in both the host and the virus. Notably, the virus was phosphorylated using the host enzyme for better survival and further mutations. Besides, SARS-CoV2 induces phosphorylation of many genes at distinct locations, and this phosphorylation is time-dependent after infection(Chatterjee and Thakur 2022, Mohapatra et al., 2022). Herein, we illustrated that SARS-CoV2 produces significant time-dependent phosphorylation of ACE2 at position s787 and y781 but not at s680 when compared with SARS-CoV and mock. As a result, metformin's phosphorylation of ACE2 at s680 appears to be useful for treatment purposes. Angiotensin converting enzyme-2 is a key component of the renin angiotensin system (RAS) axis and a key entry point for SARS-CoV-2, the dynamics of ACE2 expression in response to various extrinsic and intrinsic factors are of current interest. Therefore, understanding the ACE2 dynamic as a critical interaction between the covid-19 pandemic and other communicable and noncommunicable diseases is an attractive approach(Rath et al., 2021).In this regard, our analysis demonstrated that SARS-CoV2 infection induces significant transcription dynamics only after 6 hours. On the other hand, SARS-CoV2 infection causes ACE2 protein dynamic after 12 and 24 hours but not after 6 hours. It appears that SARS-CoV2

infection causes significant transcription dynamics of ACE2 after 6 hours, followed by protein dynamics after 12 and 24 hours.

COVID-19 can cause gastrointestinal (GI) side effects in addition to the typical systemic (fever) and respiratory (cough, dyspnea) symptoms(Ma et al., 2020). In the active phase of COVID-19, pulmonary symptoms are the hallmark of severe cases. At the same time, approximately half of the COVID-19 patients exhibit extrapulmonary GI tropism with gut clinical symptomology accompanied by virion particles shed in feces and RNA in toilet aerosols, and intestinal symptoms persist in long term post acute sequelae of SARS-CoV-2 (PASC) (Stevens et al., 2021, Almutairi et al., 2022). This is the first research to highlight metformin's potential function in modulating GI adverse effects via the ACE2-B^0AT1 complex. These elements combine to form a [ACE2:B^0AT1]2 dimer-of-heterodimer quaternary complex that is thought to direct SARS-CoV-2 tropism in the GI tract. GI clinical symptomology has been recorded in around half of COVID-19 patients, and it can be accompanied with virion particle shedding in the gut. Besides, in local gut mucosa renin–angiotensin system regulating absorption of sodium and organic nutrients(Stevens et al., 2021).

5. *Conclusions:*

Here, we presented the stability and flexibility changes of phosphorylated-ACE2-S680 in WT and variants of SARS-CoV-2 by using MD simulation. The homologous region between the phosphorylated-ACE2-S680 forms of WT and variants is high in S protein when compared with WT. However, the interaction patterns showed variation with different degrees of flexibility to induce their effect. Our findings revealed a considerable shift in all SARS-CoV-2 variants with the critical sites of the ACE2-B^0AT1 complex, with the largest fluctuations occurring in the case of omicron-ACE2-S680-Phosphorylated.These findings contribute to our understanding of metformin's potential mechanism, which will aid in future translational research aimed at treating or mitigating COVID-19 variant breakouts and/or GI symptom persistence in long-term PASC.

Figures legend

Figure 1: Phosphorylation sites in SATS-CoV2 versus mock after 6,12,24, and 36 hours.

Figure 2: Intensity of phosphorylation of ACE2-S787 in mock, SARS-COV, and SARS-CoV2 after 6, 12, and 24 hours.

Figure 3: Intensity of phosphorylation of ACE2-Y781 in mock, SARS-COV, and SARS-CoV2 after 6, 12, and 24 hours.

Figure 4: Transcription dynamic of ACE2 in mock, SARS-COV, and SARS-CoV2 after 6, 12, and 24 hours.

Figure 5: Protein dynamic of ACE2 in mock, SARS-COV, and SARS-CoV2 after 6, 12, and 24 hours.

Figure 6: Location of the most common mutations in SARS-CoV-2 S proteins: WT, delta, omicron, and gamma.

Figure 7: Comparison of the RMSD, RMSF, SASA, Rg, and number of HBs of WT, W T-ACE2-S680 phosphorylated, Delta-ACE2-S680 phosphorylated, Gamma-ACE2-S680 phosphorylated, and Omicron-ACE2-S680 phosphorylated. The results were calculated by Desmond and VMD.

Figure 8: RMSD structure superimposed at 0, 25, and 50 ns simulation periods of WT, WT-ACE2-S680 phosphorylated, Delta-ACE2-S680 phosphorylated, Gamma-ACE2-S680 phosphorylated, and Omicron-ACE2-S680 phosphorylated. PyMOL calculated the RMSD. The cycle refers to RBD (the area where the virus binds to human protein).

Figure 9: Changes in the residue index in the formation of protein SSE, such as alpha-helices and beta-strands, are characterized during the simulation times of WT, WT-ACE2-S680 phosphorylated, Delta-ACE2-S680 phosphorylated, Gamma-ACE2-S680 phosphorylated and Omicron-ACE2-S680 phosphorylated of SARS-CoV-2. The blue arrow indicates the difference. (For interpretation of the references to color in this figure legend, the reader is referred to the Web version of this article.)

Figure 10: Protein SSE such as alpha-helices and beta-strands are monitored throughout the simulation periods of WT, WT-ACE2-S680 phosphorylated, Delta-ACE2-S680 phosphorylated, Gamma-ACE2-S680 phosphorylated and Omicron-ACE2-S680 phosphorylated of SARS-CoV-2. The blue arrow indicates the difference. (For interpretation of the references to color in this figure legend, the reader is referred to the Web version of this article.)

Figure 11: The amount of change and shifting in the space of Lys625, at 2-time points 50 and 100 ns with WT, WT-ACE2-S680 phosphorylated, Delta-ACE2-S680 phosphorylated, Gamma-ACE2-S680 phosphorylated and Omicron-ACE2-S680 phosphorylated of SARS-CoV-2.

Figure 12: The amount of change and shifting in the space of Lys676, at 2-time points 50 and 100 ns with WT, WT-ACE2-S680 phosphorylated, Delta-ACE2-S680 phosphorylated, Gamma-ACE2-S680 phosphorylated and Omicron-ACE2-S680 phosphorylated of SARS-CoV-2.

Figure 13: The amount of change and shifting in the space of Arg678, at 2-time points 50 and 100 ns with WT, WT-ACE2-S680 phosphorylated, Delta-ACE2-S680 phosphorylated, Gamma-ACE2-S680 phosphorylated and Omicron-ACE2-S680 phosphorylated of SARS-CoV-2.

Molecular dynamic and bioinformatic studies of metformin-induced ACE2 phosphorylation in the presence of different SARS-CoV-2 S protein-mutations

ORIGINALITY REPORT

6%	4%	6%	1%
SIMILARITY INDEX	INTERNET SOURCES	PUBLICATIONS	STUDENT PAPERS

MATCH ALL SOURCES (ONLY SELECTED SOURCE PRINTED)

6%

★ Mohnad Abdalla, Wafa Ali Eltayb, Amr Ahmed El-Arabey, Kamal Singh, Xinyi Jiang. "Molecular dynamic study of SARS-CoV-2 with various S protein mutations and their effect on thermodynamic properties", Computers in Biology and Medicine, 2022

Publication

Exclude quotes	Off	Exclude matches	Off
Exclude bibliography	On		
